# Supplementary figures and images for: AgriSeqDB: an online RNA-Seq database for functional studies of agriculturally relevant plant species
Source: BMC Plant Biol. 2018 Sep 19;18:200. doi: 10.1186/s12870-018-1406-2 (PMC6146512; doi:10.1186/s12870-018-1406-2)

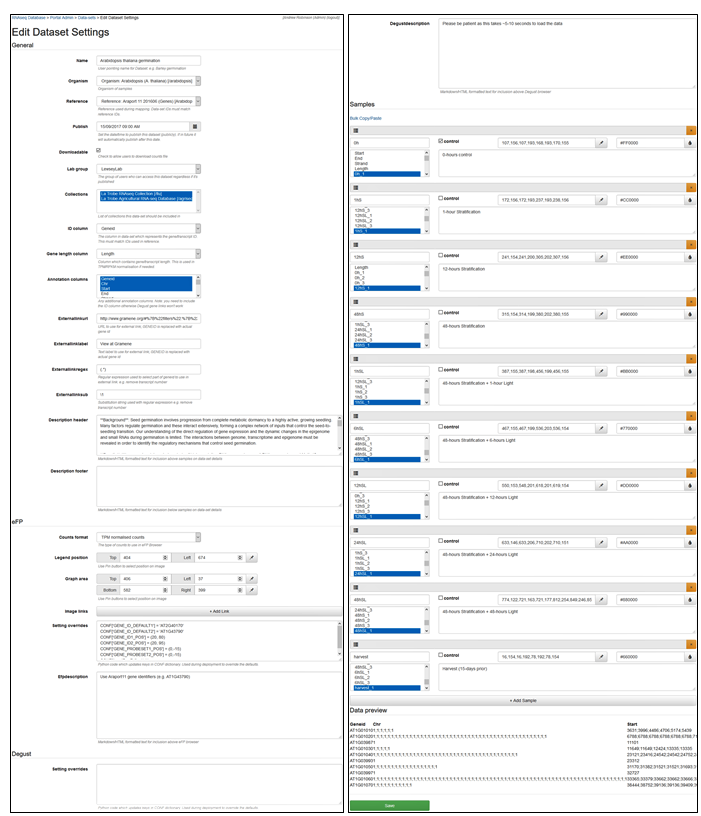

Supplement: Supplementary file 1 — Figure S1 Many of the setting the user can alter during the process of uploading a data-set to AgriSeqDB to control how the data-set is displayed in the landing portal and each data-viewer. (PNG 165 kb) [file 12870_2018_1406_MOESM1_ESM.png]
